# A real‐time PCR assay to detect predation by spiny dogfish on Atlantic cod in the western North Atlantic Ocean

**DOI:** 10.1002/ece3.6694

**Published:** 2020-09-30

**Authors:** Steven C. Pitchford, Brian E. Smith, Richard S. McBride

**Affiliations:** ^1^ Northeast Fisheries Science Center NOAA Fisheries Milford Connecticut USA; ^2^ Northeast Fisheries Science Center NOAA Fisheries Woods Hole Massachusetts USA

**Keywords:** diet analysis, fish, Fisheries Management, food webs, predator prey interactions

## Abstract

Conventional observations show spiny dogfish (*Squalus acanthius* Linnaeus) rarely eat Atlantic cod (*Gadus morhua* Linnaeus; 0.02% of stomachs) in the northwestern Atlantic Ocean. Critics express concern that digestion may limit species‐level prey identification, and with recovery from overfishing, dogfish populations may be suppressing cod by competition or predation. This study applied a real‐time PCR TaqMan assay to identify cod in dogfish stomachs collected by cooperating fishing boats during normal trawling operations (May 2014–May 2015; Gulf of Maine, Georges Bank). Conventional methods observed 51 different prey taxa and nearly 1,600 individual prey items, but no cod were observed. Cod DNA was detected in 31 (10.5%) of the dogfish stomachs, with a higher percentage of these from the homogenate of amorphous, well‐digested prey and stomach fluids (20 stomachs or 65%) than from discrete animal tissues (11 stomachs or 35%). Re‐examination of photographs of these 11 tissue samples revealed one whole, partially digested fish that could be recognized in hindsight as cod. Cod DNA was observed in dogfish stomachs year round: in January (1 of 1 trip), February (1 of 1), May (1 of 3), June (0 of 1), July (3 of 4), August (1 of 2), and October (3 of 3). Although these data suggest higher interaction rates between dogfish and cod than previously observed, addressing the population consequences of this predator–prey relationship requires a robust sampling design, estimates of digestion rates by dogfish to account for complete degradation of DNA sequences, and consideration for dogfish scavenging during fishing operations.

## Graphical abstract

This investigation leveraged samples from a cooperating fishing fleet to evaluate the feasibility of detecting Atlantic cod DNA in spiny dogfish stomachs. Detections occurred at higher rates than previously reported by traditional methods (visual inspection of stomachs). We consider necessary steps in the sampling design to robustly estimate predation rates by spiny dogfish on Atlantic cod.

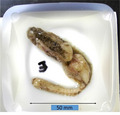

## 1 INTRODUCTION

Spiny dogfish (*Squalus acanthius* Linnaeus), after being declared overfished on the northeast U.S. continental shelf in 1998, recovered in about a decade to a higher but variable abundance (Sagarese et al., 2015). Historic diet data suggest that spiny dogfish's role as a piscivorous predator of several groundfish has not been significant (Link, Garrison, & Almeida, 2002), but a recent, simulated food web model suggests that spiny dogfish's role as a competitor and predator has changed as its population has shifted from a depleted to a rebuilt state (Morgan & Sulikowski, 2015). The focus of such debate is often whether spiny dogfish may be regulating the depleted status of Atlantic cod (*Gadus morhua* Linnaeus), a sympatric piscivore and potential prey, by either competition or predation.

The main prey in spiny dogfish stomachs are fishes, usually in a well‐digested state (Smith & Link, 2010). Among the macroscopically identifiable fish taxa, herrings (Clupeidae) and mackerels (Scombridae) dominate. Nonfish prey can at times be more prominent by geographic location, such as ctenophores on Georges Bank, or more diverse, such as to include squid and bivalves (Smith & Link, 2010). Still, cod as prey are relatively rare. Only 14 cod have been visually observed in the stomachs of 72,241 dogfish collected by fishery‐independent bottom trawl survey in the western Atlantic from 1977 to 2017 (*Northeast Fisheries Science Center, unpublished data*), which suggests low predation rates on cod. However, predation rates may appear low if the cod eaten are small and therefore more likely among the “well‐digested prey” mass, which is difficult to identify to species by macroscopic observation.

Predator–prey relations may also be examined at a molecular level from stomach contents. Although the stomach presents a highly acidic, degrading environment, a DNA‐based approach to prey identification is becoming widely used (Collier, Fitzgerald, Hice, Frisk, & McElroy, 2014; Hargrove, Parkyn, Murie, Demopoulos, & Austin, 2012; Pompanon et al., 2012). Herein, we applied a real‐time PCR TaqMan assay, initially developed by Taylor, Fox, Rico, and Rico (2002), to identify cod in dogfish stomach contents. We also adopted three steps outlined by Rosel and Kocher (2002) for cost‐effectiveness, for reduced false positives, and for completeness: (1) prescreening with specific primers to exclude nongadid samples, (2) using universal 16S primers to check for DNA quality and PCR inhibition, and (3) homogenizing unidentifiable prey before subsampling for DNA.

Our focus here was to quantify dogfish predation to assess its interaction with regional otter‐trawling fisheries. Therefore, samples of fish were collected from normal operations of commercial fishing boats in two areas: the Gulf of Maine and on Georges Bank. Such a field sampling approach has limits, because it lacked the statistical design to infer predation rates at more general spatial scales or seasonal periods. However, it begins to address a concern by industry partners that predation rates of dogfish on cod may be higher if a new sampling tool was available. To do so, the molecular results were compared to typical, macroscopic analysis of prey with the same stomach samples. We also consider the potential to apply this approach more broadly: to estimate total population consumption rates by spiny dogfish on Atlantic cod for a comparison to previous work by Link et al. (2002).

## 2 MATERIAL AND METHODS

### 2.1 Predator sampling

**FIGURE 1:**
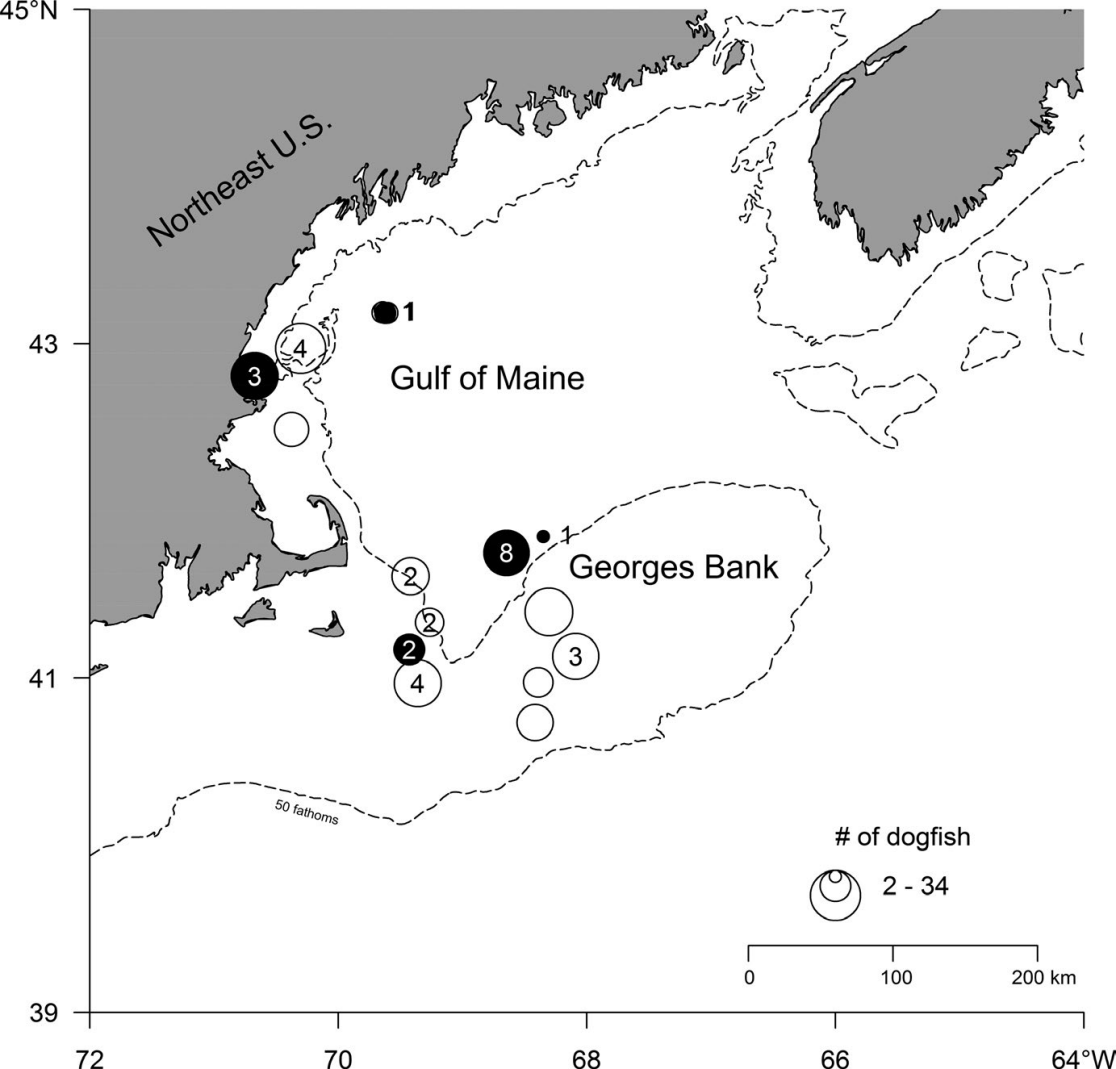
Collection locations of spiny dogfish *Squalus acanthius*. Bubble size indicates number of dogfish. Numbers within or adjacent to bubbles denote number of dogfish with positively identified Atlantic cod (*Gadus morhua*). Black bubbles indicate locations where cod fishing was actively occurring during sampling. Note, the northernmost sampling location is composed of two separate collections each with one positively identified cod and active cod fishing

Dogfish were collected on 15 trips from May 2014 to May 2015. Sampling occurred during normal, commercial otter‐trawling operations by participants of the NEFSC Cooperative Research Study Fleet in the Gulf of Maine and on Georges Bank (Figure 1). All four quarters of the year are represented, with at least one independent sampling trip in January (1), February (1), May (3), June (1), July (4), August (2), and October (3). It was noted if the trip was catching cod, as a possible source of contamination (Figure 1).

The target sample number of dogfish per trip was 30, but the realized number of fish per trip ranged from 2 to 41, totaling 295 dogfish. Such a total sample of dogfish was sufficiently large that only a single cod present would exceed the percent (1 of 295 = 0.34%) in our nominal reference sample (i.e., 14 cod in 72,241 dogfish stomachs [0.02%] from all dogfish exampled during spring and fall bottom trawl surveys, 1977–2017 [Smith & Link, 2010]). In terms of statistical power, setting aside that these fishery‐dependent versus fishery‐independent approaches employ different sampling designs (e.g., varying space and nonrandom versus stratified‐strata, random‐station approaches, respectively), power analysis showed that a threshold of only 2 cod in 295 stomachs would have been significantly more frequent than this reference (i.e., Prob. = .0014 using a glm with a Poisson probability distribution).

All samples of dogfish per fishing trip, save one, were dominated by females (total of 245 females, 50 males). Fish ranged in size from 56 to 97 cm total length. Whole dogfish were kept on ice until dissected within 24 hr.

### 2.2 Prey sampling

Stomachs were excised whole, wrapped individually in a plastic bag, and frozen (−20°C). Later, these stomachs were thawed at 4°C, carefully cut open and the total contents removed and weighed. To minimize cross‐contamination of DNA for molecular examination, nondisposable labware were soaked in 10% bleach solution, rinsed with sterile, deionized‐distilled water, and autoclaved between individual samples; dissecting tools were scrubbed and flame sterilized between samples.

Stomachs that appeared empty were gently scraped or swabbed for a DNA sample. If not, the gut contents were first examined macroscopically, identified, and separated to the lowest taxonomic level and weighed individually. Care was taken to keep identifiably individual prey whole, so it could be analyzed as a unique sample. Well‐digested prey (WDP) and any remaining stomach fluids (water, small tissues, chyme, mucous, sediment) were separated from the rest of the contents as much as possible and treated as an individual subsample. For reference, all of the remains were photographed. Invertebrates, vegetation, miscellaneous detritus, and verifiable noncod fish were excluded from further processing.

Processing of the potentially multiple sub‐samples within the stomach varied depending on whether a sample was discrete animal tissue or it was WDP or stomach fluids. Discrete animal tissue was firm and intact, such as a whole or partial fish, or pieces of bones, skin, fins etc. Possible contamination of DNA from multiple species was reduced in the final sample by either (1) preferably excising a final tissue sample from the interior of the specimen, or (2) gently scraping the tissue sample to remove surface impurities, soaking in 1%–2% bleach for 60 s, and rinsing in sterile water (Buser, Davis, Jimenez‐Hidalgo, & Hauser, 2009; Mitchell, Allister, Stick, & Hauser, 2008). Tissue samples were frozen in 2 ml cryovials at −80°C, and replicate tissues, if available were also preserved in 95% EtOH. The WDP and stomach fluids were treated separately from the discrete animal tissue described above. This included soft, amorphous, and well‐digested remains, ctenophore or ctenophore‐like soft tissues, as well as sediment, mucous, chyme, liquid, or a sample recovered from gently scraping the stomach lining. In order to not miss any target DNA (Rosel & Kocher, 2002), accumulated remains over 50 ml were pulse homogenized with a Waring style blender. A homogenizing probe (Omni Tissue Homogenizer, Omni International, Kennesaw, GA, USA) was used for smaller volumes. Aliquots were frozen in 2 ml cryovials at −80°C.

### 2.3 DNA extraction

A total of 291 dogfish stomachs were examined by molecular techniques for the presence of cod. DNA was extracted from 625 tissue and homogenized dogfish gut samples as well as 22 reference prey tissues (Table 1) for a total of 647 samples.

**TABLE 1:** Reference tissues used to test the specificity of the cod probe

Voucher prey	
Gadiformes	
Atlantic cod	*Gadus morhua*
Cusk	*Brosme brosme*
Fourbeard rockling	*Enchelyopus cimbrius*
Haddock	*Melanogrammus aeglefinus*
Pollock	*Pollachius virens*
Red hake	*Urophycis chuss*
Silver hake	*Merluccius bilinearis*
White hake	*Urophycis tenuis*
Pelagics	
Alewife	*Alosa pseudoharengus*
American shad	*Alosa sapidissima*
Atlantic herring	*Clupea harengus*
Atlantic mackerel	*Scomber scombrus*
Blueback herring	*Alosa aestivalis*
Hickory shad	*Alosa mediocris*
Sand lance	*Ammodytes* sp
Butterfish	*Peprilus triacanthus*
Other	
Ctenophores	*Ctenophora*
Illex squid	*Illex* sp
Loligo squid	*Loligo* sp
Ocean pout	*Macrozoarces americanus*
Scup	*Stenotomus chrysops*
Spiny dogfish	*Squalus acanthias*

Extraction of the DNA from samples of discrete animal tissues used the salt precipitation method of Aljanabi and Martinez (1997) or the QIAamp Mini Kit (Qiagen Hilden, Germany). For the homogenized and stomach scrapings (also homogenized), the QIAamp Fast DNA Stool Mini Kit (Qiagen) was used.

Quantification and purity of the extracted DNA was measured spectrophotometrically with a Nanodrop 2000 and the concentrations were adjusted to 50–100 ng/µl. The DNA samples were stored at −20°C.

### 2.4 DNA quality and gadid screening

Stomachs are highly acidic and degenerating environments for tissues and nucleic acids. Therefore an important step before further processing is to determine the usability of the extracted DNA. Amplification of DNA from all samples used conventional PCR (cnPCR) (Bastien, Procop, & Reischl, 2008). Universal mitochondrial 16S rRNA gene primers (Forward 16sar‐L ‐5ʹCGC‐CTG‐TTT‐ATC‐AAA‐AAC‐AT‐3ʹ; Reverse 16sar‐H 5ʹ CCG‐GTC‐TGA‐ACT‐CAG‐ATC‐ACG‐T 3ʹ (Palumbi et al., 2002)) were employed to check the quality, inhibition, and the presence of amplifiable DNA (Ivanova, Zemlak, Hanner, & Hebert, 2007; Rosel & Kocher, 2002). The expected size of the amplicons ranged from 500–650 bp. The cnPCR volumes were 25 µl with 12.5 µl One Taq Quick‐Load 2× Master Mix (New England Biolabs), 0.5 µl each primer (50 µMol) (IDT), 1 µl sterile nuclease‐free water (Hyclone), 31.25 mg BSA (New England Biolabs) and 50 ng of DNA template. Cycling conditions included an initial denaturation at 94°C for 2 min, 35 cycles of denaturation, annealing, extension: (94°C for 30 s, 50°C for 40 s, 72°C for 60 s), a final extension at 72°C for 10 min, followed by 4°C hold. Amplicons were visualized with 2% agarose gel stained by ethidium bromide.

In order to narrow the focus to cod and cod‐like prey, all sample DNA was screened to select only the gadid‐related remains for subsequent real‐time PCR (King, Read, Traugott, & Symondson, 2008; Rosel & Kocher, 2002). The screening used a cnPCR procedure adapted from Taylor et al. (2002), where a short, 103 bp region of the ATP synthase subunit six (ATPase6) and eight (ATPase8) genes of cod was amplified; Gadid Forward: 5ʹ GCA ATC GAG TTG TAT CTC TAC AAG GAT 3ʹ and Gadid Reverse: 5ʹ CAC AAA TGA GCT CCT CTT CTT GC 3ʹ. Since this target is much shorter than the 16S rRNA gene (500–650 bp) and perhaps more likely to be encountered in degraded samples, the few samples negative (3.4%; Table 2) from the previous step were also included. The cnPCR mix was the same as described for the 16S quality check. Cycling conditions included an initial denaturation at 94°C for 3 min, 35 cycles of denaturation, annealing, extension: (94°C for 30 s, 58°C for 30 s, 68°C for 30 s), a final extension at 68°C for 5 min, followed by 4°C hold. The PCR amplicons were visualized with 3% agarose gel stained by ethidium bromide. Only those samples producing a single 103 bp band were used in real‐time PCR assays.

**TABLE 2:** Summary of molecular results for both the conventional and real‐time PCR

	Number of positive samples/assay				
	Reference Prey (*n* = 22)	Tissues (T) (*n* = 336)	Homogenized (H) (*n* = 289)	Total (T + H) (*n* = 625)	Stomachs (*n* = 291)
16S cnPCR	22	318	286	604	278
Gadidae cnPCR	4/22^a^	143	98	241	107
qPCR Round 1	1/22 (cod only)	26	32	58	40
qPCR Round 2		11	20	31	31

^a^ Four species positive for cnPCR with gadid primers: cod, haddock, pollock, cusk.

### 2.5 Real‐time PCR

Real‐time PCR assays and analysis were performed on an Applied Biosystems StepOnePlus™ Real Time PCR system using the Presence/Absence module (Applied Biosystems StepOne™ and StepOnePlus™ Real‐Time PCR Systems Getting Started Guide for Presence/Absence Experiments Part Number 4376787 Rev. E 06/2010). The primers used for the gadid screening step were also used for the Taqman^®^ assays. The labeled MGB Taqman^®^ cod probe with Non Fluorescent Quencher (6‐FAM CTT‐TTT‐ACC‐TCT‐AAA‐TGT‐GGG‐AGG MGB‐NFQ) was originally developed by M.J. Taylor (Taylor et al., 2002). Potential cross‐reactions were ruled out by checking the primer pair and probe GenBank using Primer‐BLAST and BLASTn.

The specificity of the cod‐specific TaqMan probe was checked against reference tissues commonly found in dogfish stomachs (Smith & Link, 2010). All species used for the specificity tests were collected during a spring, 2014, groundfish survey conducted by the Northeast Fisheries Science Center, except for a cusk sample from a 2015 long line survey and a fourbeard rockling from a 2015 spring groundfish survey (Table 1). Real‐time PCR assays with the cod‐specific TaqMan assay were performed with all 22 reference prey. Only the cod voucher DNA produced a positive signal (Table 2). Subsequently, the same cod DNA was also used as the positive control for all real‐time assays.

Reactions were conducted in a final volume of 20 μl containing 1 μl DNA template, 1× Taqman^®^ Universal PCR Mastermix, II with AmpErase^®^ uracil N‐glycosylase (UNG) (Applied Biosystems), 250 nM Taqman^®^ probe (Applied Biosystems), 300 nM of each primer (IDT), 1.25 mg/ul BSA (NEB) and nuclease‐free water (HyClone). Assays were performed using the recommended cycling program: 50°C for 2 min (UNG incubation), 95°C for 10 min (polymerase activation), followed by 45 cycles of 95°C for 15 s (denaturation) and 60°C for 1 min (annealing/extension). All samples, the positive controls (cod DNA) and no template negative controls were run in triplicate in sealed 96 well plates (Applied Biosystems). To rule out PCR failure or false negatives due to lack of target, contaminants, or inhibitors, all reactions included the Taqman^®^ IPC.

Initially, some tissue and most homogenate real‐time reactions produced unconfirmed calls, indicating failure of the IPC. A short series of experiments were performed to try to alleviate these failures. Unsuccessful IPC reactions were rerun with the addition 1.25 µg/µl Bovine Serum Albumin (BSA) (New England Biolabs) (Albaina et al., 2010; Juen & Traugott, 2006). Ten‐fold serial dilutions (5.0, 0.05, 0.005 ng/µl) of three homogenized sample DNA with and without BSA was also performed. The addition of BSA without further diluting the template was found to be the optimum treatment and was used for all of the subsequent real‐time TaqMan assays (Table 3).

**TABLE 3:** Results of the BSA and BSA/dilution tests to eliminate failures of the IPC. These failures led to unconfirmed calls in individual real‐time PCR wells. All samples were run in triplicate, but a few had both positive and negative reactions. Final concentration of added BSA was 1.25 µg/µl. (ND: Tissue DNA dilutions were not done)

	Tissues		Homogenates	
	IPC‐positive reactions	IPC‐negative reactions	IPC‐positive reactions	IPC‐negative reactions
Before addition of BSA	172 (89.1%)	21 (10.9%)	17 (29.3%)	41 (70.7%)
After BSA added to IPC‐negative reactions	15 (100%)	0	45 (100%)	0
Dilution with and without BSA				
Diluted/(−) BSA	ND	ND	9 (33.3%)	18 (66.7%)
Diluted/(+) BSA	ND	ND	27 (100%)	0
Remainder of samples				
BSA added	435 (100%)	0	333 (100%)	0

To determine the efficiency of the real‐time primers and probe, a standard curve of 10 fold serial dilutions of cod DNA was tested. Each dilution was replicated six times. A standard curve of the mean cycle threshold (*C_t_*) values versus concentration of cod DNA was produced and the slope and *R* ^2^ values determine the efficiency of the reactions (Figure 4). Unlike some other real‐time or qPCR Taqman assays, standard curves or *C_t_* amplification plots are not used to make the presence/absence calls. Instead, calls were based on the difference between the Negative Control‐IPC wells and the relative fluorescent intensity at the end of 40 cycles and calculated by the instrument software. The *C_t_* values used to calculate the standard curve are provided as part of the results.

Two rounds of assays were performed with the gadid positive samples. In the first round, a sample was considered positive for cod when 2/3 or 3/3 replicates were called “Present” with a confidence level of at least 99.0%. For the 2nd round, the assays were again repeated in triplicate but only with the samples having 2/3 or 3/3 positive wells. If there were multiple positive samples from the same stomach in the 1st round, only the one with the highest positive signal was repeated. A sample was considered positive for cod when at least 5/6 wells from both the rounds were called “Present” with confidence levels of at least 99.0%.

## 3 RESULTS

### 3.1 Macroscopic examination

**FIGURE 2:**
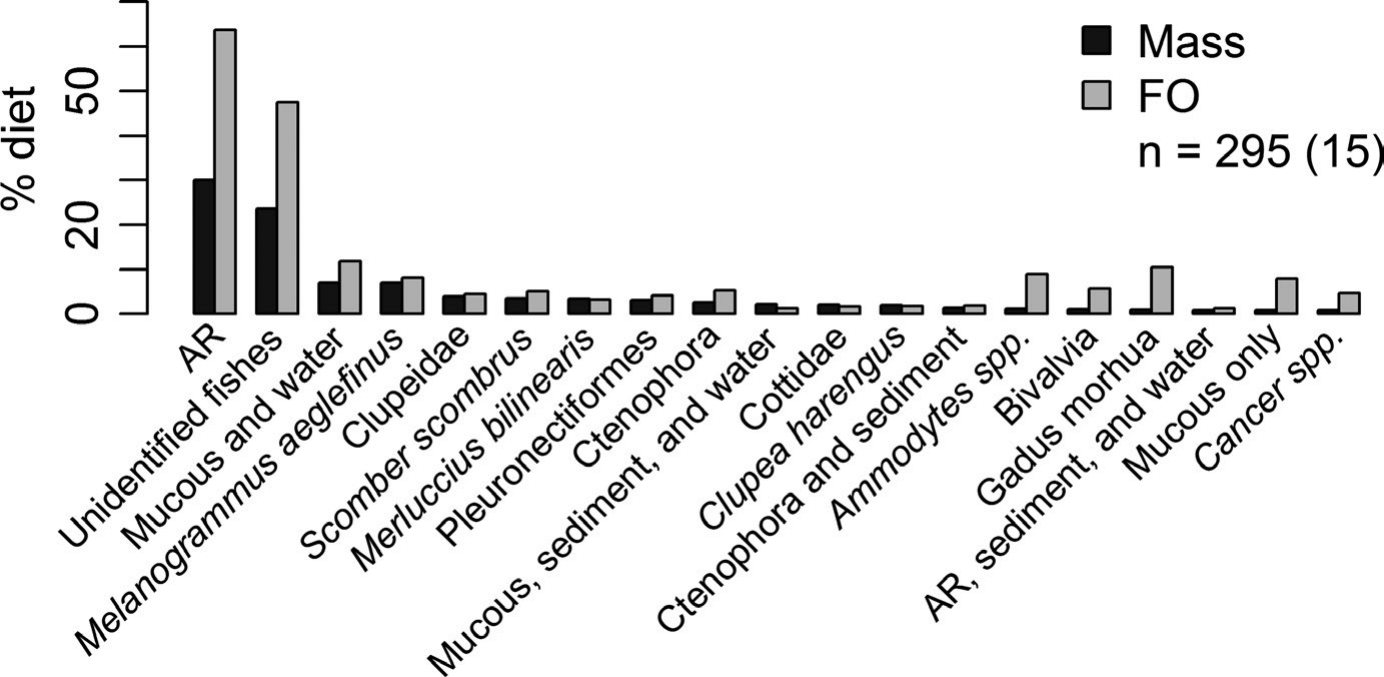
Percent mass (Mass) and frequency of occurrence (FO) of major prey species and groups for spiny dogfish *Squalus acanthius* identified by macroscopic and molecular examination. The number of stomachs, n, and the number of tows in parentheses are provided. Unidentifiable animal remains denoted by “AR.”

In aggregate, 295 dogfish yielded 35.53 kg of stomach contents, comprised of 51 different prey taxa and nearly 1,600 individual prey items (Figure 2). By weight, the majority of prey taxa were unidentified and were treated as either individual items of well‐digested prey (8.3 kg; 23.4%) or homogenized samples (14.7 kg; 41.3%). Nearly all (90.0% by weight) of the 336 tissues subsamples were fish or “fish‐like” pieces of firm and detached flesh; seven percent (by weight) could not be distinguished between vertebrate or invertebrate tissue, and 3% were ctenophore‐like or miscellaneous but unknown. The composition of the 289 homogenized subsamples was WDP (69%), mucous or mucous and water (24%), and ctenophora or “ctenophora‐like” tissue (7%).

**FIGURE 3:**
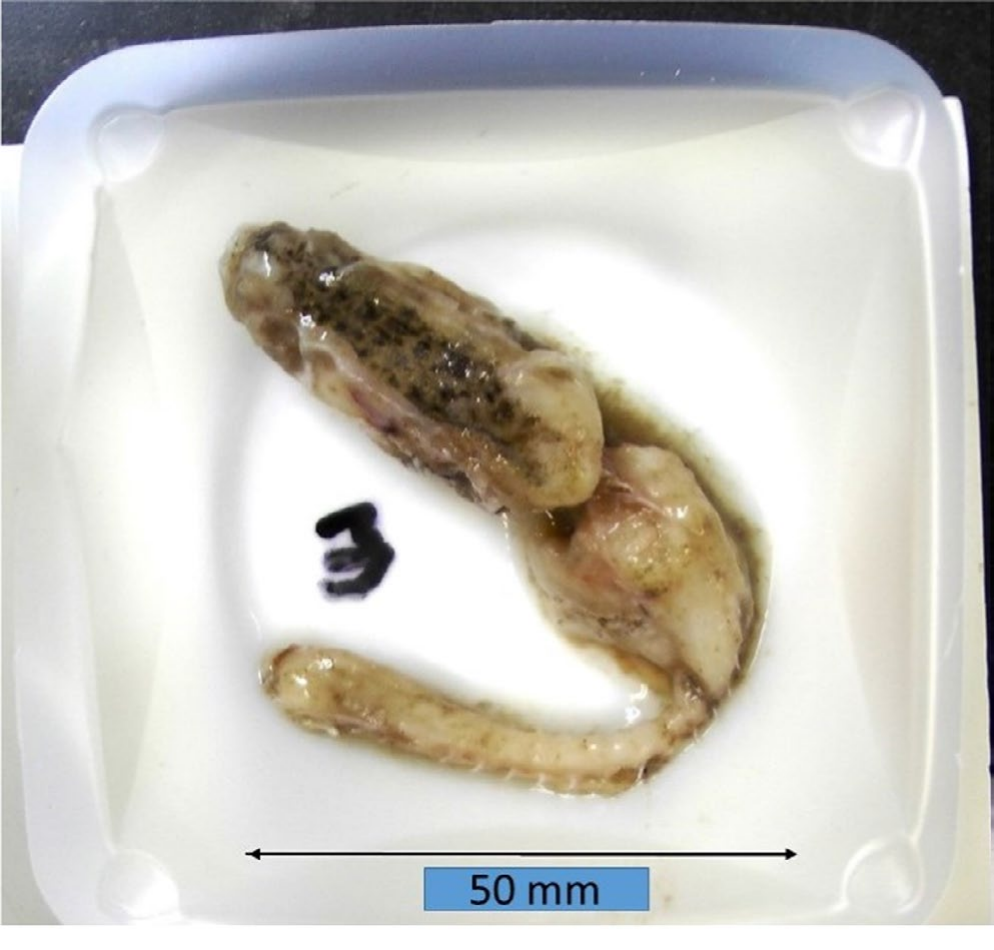
Initially classified as unidentified fish remains, real‐time PCR identified this as Atlantic cod (*Gadus morhua*). The mottled pattern on head is characteristic of juvenile cod

Unidentifiable prey comprised 64.7% and identifiable prey 35.3% of the total biomass recovered from dogfish stomachs. Of the identifiable prey, most were fish (92% by weight), with haddock (*Melanogrammus aeglefinus*) being the single highest species (29% of the total identifiable prey). Other notable identifiable prey were crustaceans (5%) and bivalves (2.1%). After completion of the macroscopic examinations, no remains resembling cod had been observed. However, a well‐digested juvenile fish produced a strong positive reaction with the cod probe. After consulting the photo of the fish, portions of the head still retained a mottled skin pattern characteristic of cod juveniles (Figure 3).

### 3.2 Molecular results

cnPCR with universal mitochondrial 16S rRNA gene primers successfully amplified samples that were not ruled out as noncod by macroscopic identification, including all 22 reference prey species, 94.64% (318/336) of the discrete but unidentified animal tissues and 98.96% (286/289) of the homogenized samples. cnPCR of the 22 reference prey species using the gadid primers produced the expected band at 103bp not only for cod, but also for three other closely related species: haddock, pollock, and cusk. About a third (107/291) of the stomachs examined and 38.6% (241/625) of the unidentifiable prey samples tested positive for at least one of these four gadid species.

All 241 positive samples from the gadid screening step were analyzed by real‐time PCR for the presence of cod. Cod DNA was found in 31 of 295 (10.5%) of the dogfish stomachs, with a higher percentage of these from the homogenized (65%) compared to the tissue subsamples (35%) (real‐time PCR round 2; Table 2). Cod DNA was observed in dogfish stomachs from all four quarters of the year, in January (1 of 1 trip), February (1 of 1), May (1 of 3), June (0 of 1), July (3 of 4), August (1 of 2), and October (3 of 3).

Results of trials to eliminate failures of the TaqMan Exogenous Internal Positive Control (IPC) in some of the initial reactions are given in Table 3. IPC failure is usually the indication of deleterious substances preventing or hampering amplification of both the IPC and templates. IPC failures were much higher in the early runs of the homogenates (70.7%) compared to the tissue (10.9%). However, when IPC‐negative tissue and homogenate sample DNA were rerun with the addition of BSA, 100% of the IPC reactions turned positive. Diluting the homogenate templates, a proven method in other studies (King 2008), was inconclusive. Three samples were diluted with and without the addition of 1.25 µg/µl BSA. Without BSA, one of three samples was IPC positive for all reactions. For the other two samples, all dilutions were IPC negative. When BSA was added, all 27 reactions in the three dilution series were IPC positive.

In view of these results, BSA was added to all tissue and homogenate real‐time reactions without diluting the templates. None of the subsequent reactions resulted in failure of the IPC. (Table 3).

**FIGURE 4:**
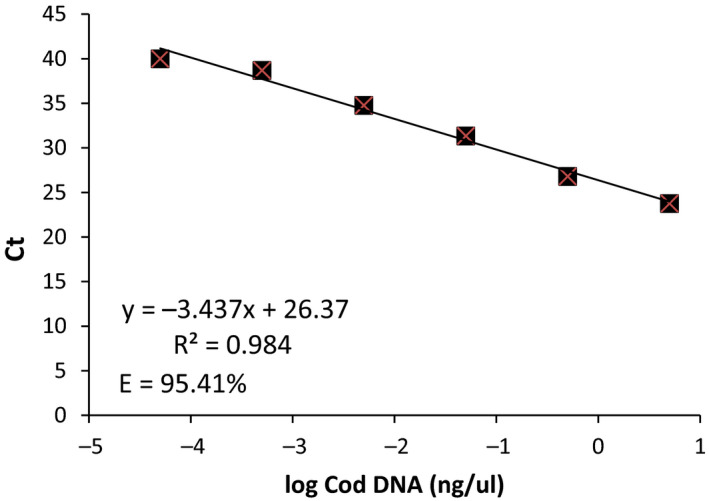
Standard curve of the probe efficiency in the real‐time Taqman assays. A 10‐fold serial dilution series of cod DNA is plotted against the cycle threshold (*C_t_*) values. Each dilution value is the mean of six replicates; error bars are smaller than the square symbols

A high correlation (*R* ^2^ = .98) was evident between the dilution factor and C_t_ (Figure 4). In addition, the slope produced a very high primer‐probe efficiency of 95.41%.

## 4 DISCUSSION

There are likely multiple reasons that we observed higher frequencies of cod in dogfish stomachs in these fishery‐dependent collections compared to our reference frequencies observed in the fishery‐independent bottom trawl survey. First, we anticipated that some cod could be small, well‐digested, or both, which would cause them to be overlooked from macroscopic identification used on the bottom trawl survey. We report a single instance of this occurring in the samples we examined here. Second, trace amounts of cod DNA may linger not just in a well‐digested mass in the stomach, but in the stomach chyme or mucus, or possibly in the stomach of a prey item eaten by the dogfish, any of which may be detected by the sensitive real‐time PCR assay employed in this study. These possibilities increase the period of detectability further, which increases the detection rate per feeding event. There may be stage‐specific or species‐specific digestion rates. For example, Rosel and Kocher (2002) report detection rates of about 12 hr from a small trial of larval cod fed to Atlantic mackerel, *Scomber scombrus*, and Hunter, Taylor, Fox, Maillard, and Taylor (2012) report >24‐hr half‐life detection rates of cod eggs and larvae fed to whiting, *Merlangius merlangus*. Digestion rates of larger prey fed to bony fishes (teleosts) are >24 hr (Buckel & Conover, 1996; Butler, Rudershausen, & Buckel, 2010) and longer still for sharks (Bangley & Rulifson, 2014; Beamish, Thomson, & McFarlane, 1992; Medved, Stillwell, & Casey, 1988), dependent on temperature (Stehlik, Phelan, Rosendale, & Hare, 2015). Determining an actual feeding rate requires knowledge of the persistence of DNA in dogfish stomachs at a range of temperatures. A third reason that could lead to higher rates of detection in our study is because fishermen report interactions between both species during operations, and while some of these interactions may be active foraging of live prey, some are not. Rafferty, Brazer, and Reina (2012) observed that 3% of cod landed from Georges Bank gillnet fishery was discarded due to spiny dogfish depredation. From the current study, 50% of the sampling trips with cod positively eaten also included observations of fish entrails and potentially discarded fish; thus, scavenging by spiny dogfish was actively occurring, as has been suspected (Hanchet, 1991; Smith, Ford, & Link, 2016), and should be accounted for in future studies. This behavior and the knowledge of dogfish sampling co‐occurring with cod fishing for a subset of the fishing trips used by this study increased our likelihood of positively detecting cod, as anticipated, but it also confounded our ability to expand our results to population‐level estimates of cod mortality.

Fishermen see dogfish associated with cod on their fishing hotspots, during a period of rebuilding dogfish and depleted cod. Low levels of Atlantic cod in the western Atlantic Ocean have been largely attributed to overfishing (Kess et al., 2019; Palmer, Deroba, Legault, & Brooks, 2016). However, regulations designed to control fishing have not led to rebuilding cod populations (Kritzer, Costello, Mangin, & Smith, 2019). Other factors that may be holding back population rebounds of cod include disruption of spawning aggregations (Armstrong et al., 2013; Dean, Hoffman, & Armstrong, 2012; Dean, Hoffman, Zemeckis, & Armstrong, 2014), warming trends that reduce habitat suitability (Adams et al., 2018; Kritzer et al., 2019), predation by planktivores such as Atlantic herring, *Clupea harengus*, or Atlantic mackerel (Bakun, 2006), and as addressed here, increasing numbers of piscivorous predators, such as spiny dogfish.

Sagarese et al. (2015) note increasing overlap, as dogfish have recovered, between the distributions of dogfish and the operations of bottom trawl and gillnet fisheries. Here, we leverage this to our advantage, because it should increase the likelihood for observing cod DNA in dogfish stomachs, aiding the development of this approach. A next step is to employ a statistically robust sampling design to examine a population‐level assessment of the effects of dogfish predation on cod population size, either with cooperating fishing boats or a fishery‐independent platform. The existing NEFSC bottom trawl survey has specific advantages because it is already operational, it covers nearly the entire geographic range of dogfish, and it is the source that generated the existing reference consumption rates (Link et al., 2002; Smith & Link, 2010). A robust spatial sampling design would benefit by partnering with other trawl surveys (e.g., the state of Massachusetts) that sample into nearshore areas not sampled well by the NEFSC survey. Experience in this pilot phase worked out the process with existing methods. Collateral research is also needed to determine detection rates of cod DNA in relation to dogfish digestion rates, and detection biases due to scavenging behavior to accurately quantify cod predation. Additionally, new technologies may increase the operational success of these molecular approaches, such as droplet digital PCR, next‐generation sequencing and multilocus metabarcoding (Albaina, Aguirre, Abad, Santos, & Estonba, 2016; Bessey et al., 2019; Bowser, Diamond, & Addison, 2013; Waraniak, Marsh, & Scribner, 2019).

## CONFLICT OF INTEREST

None declared.

## AUTHOR CONTRIBUTION

**Steven C. Pitchford:** Conceptualization (equal); Investigation (equal); Methodology (lead); Resources (equal); Writing‐original draft (equal); Writing‐review & editing (equal). **Brian E. Smith:** Conceptualization (equal); Data curation (equal); Formal analysis (equal); Writing‐original draft (equal); Writing‐review & editing (equal). **Richard S. McBride:** Conceptualization (equal); Funding acquisition (lead); Project administration (lead); Resources (lead); Supervision (lead); Writing‐original draft (equal); Writing‐review & editing (equal).

## Data Availability

The data are fully accessible on Dryad (https://doi.org/10.5061/dryad.8w9ghx3jv).
